# Circulating miRNAs as potential non-invasive biomarkers for ANCA-associated glomerulonephritis

**DOI:** 10.3389/fimmu.2025.1599043

**Published:** 2025-07-17

**Authors:** Matic Bošnjak, Željka Večerić-Haler, Živa Pipan Tkalec, Jakob Tomšič, Emanuela Boštjančič, Nika Kojc

**Affiliations:** ^1^ Institute of Pathology, Faculty of Medicine, University of Ljubljana, Ljubljana, Slovenia; ^2^ Department of Nephrology, University Medical Centre Ljubljana, Ljubljana, Slovenia; ^3^ Faculty of Medicine, University of Ljubljana, Ljubljana, Slovenia

**Keywords:** ANCA, biomarker, glomerulonephritis, microRNA, vasculitis, epigenetics

## Abstract

**Introduction:**

Anti-neutrophil cytoplasmic antibody (ANCA)-associated vasculitis (AAV) is characterized by necrotizing small vessel vasculitis that frequently manifests as glomerulonephritis (AAV-GN). An accurate noninvasive biomarker reflecting active AAV-GN remains elusive. The knowledge of microRNAs (miRNAs), which have been considered as disease-specific biomarkers, is scarce and lacks validated data in AAV.

**Methods:**

This study validated a renal tissue expression profile of candidate miRNAs specific to AAV-GN selected through prior screening using independent cohorts. The analysis was extended to serum samples to explore the potential of a circulating miRNA panel as a noninvasive biomarker for active AAV-GN. To substantiate the findings, we correlated the molecular data with clinical and histologic markers of AAV-GN activity.

**Results:**

We identified *miR-21-3p, miR-181a-5p*, and *miR-181d-5p* as potential biomarkers distinguishing AAV-GN from non-AAV renal diseases and healthy controls. In addition, *miR-21-3p* and *miR-181d-5p* correlated with the presence of active AAV-GN, while *miR-181a-5p* differentiated AAV-GN subtypes based on ANCA antigen specificity. ROC curve analysis demonstrated that the combined serum expression of *miR-21-3p* and *miR-181a-5p* reliably distinguished AAV-GN from other renal pathologies, including ANCA-positive cases without histologic evidence of AAV-GN.

**Conclusion:**

Our findings highlight the potential of circulating miRNA expression signature as a noninvasive biomarker for ongoing AAV-GN in the appropriate setting. Larger confirmatory studies are essential to support the clinical application of miRNA-based biomarkers in AAV-GN diagnostics and disease monitoring.

## 1 Introduction

Anti-neutrophil cytoplasmic antibody (ANCA)-associated vasculitis (AAV) encompasses a heterogeneous disease group characterized by autoimmune necrotizing small vessel vasculitis. A severe type of renal involvement is common in AAV, known as relapsing pauci-immune crescentic and necrotizing glomerulonephritis (AAV-GN). AAV-GN is an important determinant of disease-specific morbidity and mortality. While detectable ANCAs provide some correlation with ongoing renal involvement, the ‘ANCA-centric’ approach has limitations, including the presence of ANCAs in non-AAV diseases and the fact that rising titers do not always relate to AAV-GN activity/flare (1). An accurate noninvasive biomarker reflecting active AAV-GN thus remains elusive. Consequently, a renal biopsy, an invasive procedure with inherent risks, remains the diagnostic and prognostic gold standard in cases clinically suspected of AAV-GN.

Due to observed phenotypic and genotypic differences, AAV is best classified by the antigen specificity of ANCAs. The two primary antigens of pathogenic ANCAs are myeloperoxidase (MPO) and proteinase-3 (PR3). Notably, MPO-AAV and PR3-AAV are increasingly considered as entities with distinct pathogeneses, clinical features and management strategies (2, 3).

Altered macrophage polarization (4) and differentiation of CD4+ T-cells towards Th17 subsets (5) are two recognized biological processes implicated in the pathogenesis of AAV; however, the precise mechanisms driving their activation remain poorly understood. Epigenetic regulation through microRNAs (miRNAs) has been implicated in both macrophage polarization (6) and Th17 differentiation (7), but not in the specific context of AAV-GN. While miRNAs have been proposed as candidate biomarkers in various autoimmune diseases (8), validated data in AAV-GN are lacking. This study aimed to validate a renal miRNA expression profile identified as specific to AAV-GN (9) and investigate its potential as a circulating biomarker for active AAV-GN. Additionally, it aimed to identify miRNA expression differences between MPO- and PR3-positive AAG-GN cases and correlate the molecular data with clinical and histologic markers of disease activity to improve diagnostic accuracy.

## 2 Materials and methods

### 2.1 Study design

Matched renal tissue and serum samples were collected from 60 consecutive patients of AAV with biopsy-proven renal involvement in the form of AAV-GN, and from 28 individuals with non-AAV medical renal disease (non-AAV RD) between 2018 and 2023. Samples obtained during this period were included in analysis only if considered technically adequate for further processing after sample quality control (see **Supplementary Materials & Methods**). All AAV-GN cases demonstrated positive serological assays for ANCA (either MPO- or PR3-ANCA) on ELISA, which further stratified this group into MPO- and PR3-positive AAV-GN. The non-AAV RD group comprised a variety of inflammatory and non-inflammatory renal pathologies without detectable ANCA. A further 11 cases with positive MPO- or PR3-ANCA without clinical or histologic evidence of AAV-GN were included in the third group, referred to as the bystander ANCA group. The full list of diagnostic entities comprising the groups and the list of recorded clinical and histologic variables are available in **Supplementary Materials & Methods**.

All renal biopsies were performed at disease presentation and contained at least seven glomeruli and one interlobular-type artery to eliminate the potential confounding effect of suboptimal specimens. Matched serum samples were collected at the time of renal biopsy for ANCA serological assays by ELISA. All serum samples were snap-frozen and stored at –80°C within 72 hours of collection.

Clinical variables recorded for each case included age, gender, estimated glomerular filtration rate (eGFR in ml/min/1.73 m2, calculated using the CKD-EPI 2021 equation), ANCA serology, and measured daily proteinuria (24h urine total protein excretion in grams). For AAV-GN cases specifically, we documented the Birmingham Vasculitis Activity Score (BVAS) (10), the disease phenotype (renal limited or systemic), the presence of rapidly progressive glomerulonephritis (RPGN), the presence of pulmonary-renal syndrome, the extent of organ involvement (as total number of organs affected), and the application of pre-emptive immunosuppressive therapy and plasmapheresis at or prior to renal biopsy.

For each case, the following histologic variables were recorded: percentage of global glomerulosclerosis, extent of interstitial fibrosis/tubular atrophy in 5% increments and the presence of acute tubular injury. AAV-GN cases were classified according to two established scoring systems in AAV-GN i.e., histologic class per ‘Histopathologic classification of ANCA-associated glomerulonephritis (11)’ (Berden class) and ‘ANCA Kidney Risk Score (12)’ (AKRiS) were assigned. Additional histologic variables recorded in AAV-GN cases included the percentages of normal glomeruli (%NG) and cellular or fibrocellular glomerular crescents (%C), and the presence of medullary angiitis and arteritis in interlobular/arcuate-type arteries.

To enhance the specificity of our study at the serologic level, we included unmatched serum-only samples from 20 individuals without clinical or laboratory evidence of kidney disease and with negative ANCA serology, designated as healthy controls group (HC).

### 2.2 Total RNA isolation, reverse transcription and qPCR

Four 10-µm thick sections were cut from the formalin-fixed paraffin-embedded (FFPE) renal biopsy tissue blocks for RNA isolation into 2.0-ml microcentrifuge sterile tubes. Total RNA isolation was performed using a semi-automatic procedure with the Maxwell RSC (Promega, Madison, WI 53711-5399, USA), in combination with the Maxwell^®^ RSC RNA FFPE kit (Promega, Madison, WI 53711-5399, USA; code AS1440) and Maxwell^®^ RSC miRNA tissue kit (Promega, Madison, WI 53711-5399, USA; code AS1460).

For total RNA isolation from serum samples, 200 µl of stored serum was processed with miRNeasy Serum/Plasma Advanced Kit (Qiagen, Hilden, Germany).

See **Supplementary Materials & Methods** for detailed protocols of isolation, reverse transcription, reference genes (RGs) selection, and quantification by quantitative polymerase chain reaction. (qPCR).

### 2.3 Selection of candidate miRNAs for validation in renal tissue and serum samples

We identified 30 candidate miRNAs by (preceding) screenings using miRCURY LNA miRNA miRNOME panel (Qiagen, Hilden, Germany): 17 miRNAs that potentially distinguish AAV-GN from non-AAV GN controls and living donors (9), and 13 additional miRNAs that potentially distinguish MPO- and PR3-positive AAV-GN (the latter are presented in Results).

Candidate miRNAs from the two screening phases were selected for validation based on following criteria: (1) Cq values ≤ 30; (ii) candidate miRNAs belonging to the same miRNA family were included regardless of their Cq values; (iii) miRNAs from the three represented miRNA families that had not been statistically selected were also included for validation. The 21 miRNAs selected for validation in renal tissue samples and their specific inclusion criteria are presented in **Supplementary Table S1**. The selection process of candidate miRNAs for validation is described in more detail in **Supplementary Materials & Methods**.

Serum-based analysis and all correlations included candidate miRNAs with significant expression differences in the renal tissue between AAV-GN and non-AAV RD, MPO- and PR3-positive AAV-GN, and/or AAV-GN and bystander ANCA samples.

### 2.4 Technical yield and selection of samples included in the validation

The technical procedure (isolation and subsequent RT/qPCR) integrity was assessed by expression analysis of selected RG miRNAs in the renal tissue samples and of control spike-in RNAs in the serum samples as suggested by the manufacturer. In the renal tissue samples, comparable expression levels of RG miRNAs were identified in 57/60 AAV-GN, 26/28 non-AAV RD and 11/11 bystander ANCA samples. In the matched serum samples, the isolation and quantification of spike-in RNAs was successful in 38 of 57 AAV-GN, in 23 of 26 non-AAV RD and 6 of 11 bystander ANCA samples. In the unmatched serum samples of HC group, isolation and quantification of spike-in RNAs was successful in all 20 included samples.

### 2.5 Statistical analysis of miRNA expression and clinicopathologic characteristics

ΔCq was calculated to quantify the expression of individual miRNA gene relative to the geometric expression mean of RGs (13). The Kruskal-Wallis, Mann-Whitney, Chi-square, and Fischer’s exact tests were employed to analyze differences in ΔCq and in clinical and histologic characteristics. The Spearman’s rank correlation coefficient (Spearman’s rho) was used for all correlations, including calculation of statistical power and estimation of needed sample size. The receiver operating characteristic (ROC) curve analysis included the most promising validated serum-expressed miRNAs. All statistical analyses were performed using SPSS analytical software (IBM SPSS statistics, version 27.0, Armonk, NY, USA) with p-values < 0.05 considered statistically significant. Note that due to the nature of ΔCq value calculation, negative correlations in ΔCq values correspond to positive correlations in actual expression levels, and vice versa. See **Supplementary Materials & Methods** for detailed description of ΔCq calculation and its correlations and ROC curve analyses.

## 3 Results

**Figure 1:**
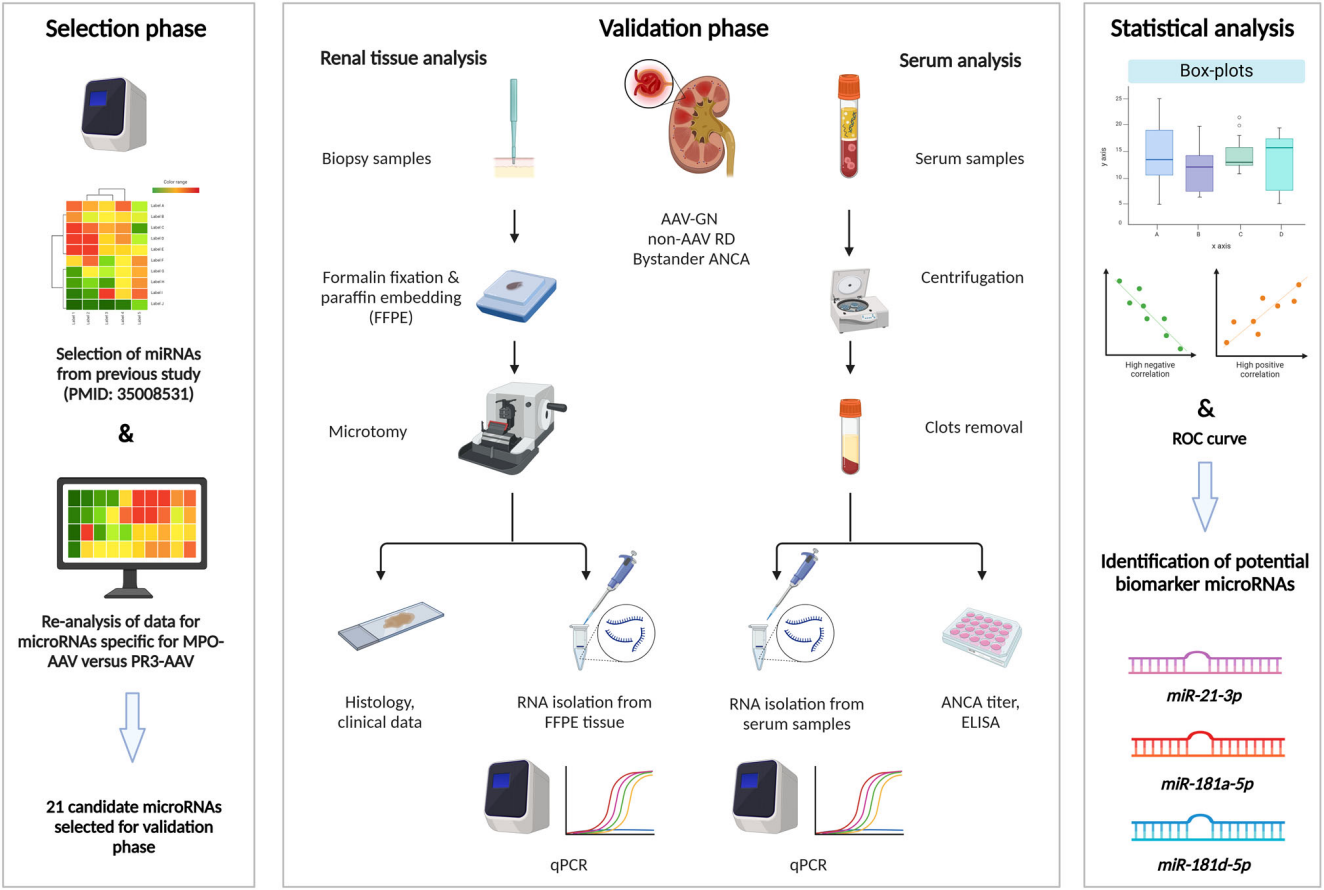
Research workflow scheme. Left: screening was based on qPCR in pooled tissue samples of AAV-GN compared to non-AAV RD and individuals without renal disease. Additional screening was performed within the AAV-GN group to identify candidate miRNAs that could distinguish MPO- from PR3-positive AAV-GN. After the statistical analysis of screening data, 21 candidate miRNAs were selected for validation. Middle: parallel analysis of expression of selected miRNAs was performed in matched renal biopsy and serum samples collected at the time of biopsy. We included 60 AAV-GN, 28 non-AAV RD and 11 bystander ANCA samples within the 2018–2023 timeframe. Fifty-seven tissue and 38 serum samples of AAV-GN, 26 tissue and 23 serum samples of non-AAV, and 11 tissue and 6 serum samples of bystander ANCA were analytically accepted after sample quality control. Validation was performed using qPCR. Right: The three most promising miRNAs were determined through statistical analysis of differential tissue and serum expression and through correlations to scoring systems for renal involvement and clinical variables of disease activity in AAV-GN. AAV-GN, patients with ANCA-associated glomerulonephritis; ANCA, anti-neutrophil cytoplasmic antibody; ELISA, enzyme-linked immunosorbent assay; non-AAV RD, non-AAV medical renal disease controls; qPCR, quantitative polymerase chain reaction.

A schematic workflow of this research is depicted in **Figure 1**. Briefly, we identified 17 candidate miRNAs that potentially distinguish AAV-GN as a group from non-AAV GN controls, as well as from individuals without renal disease (living donors) in the previous study (9). In this study, we performed a similar analysis of screening data and identified 13 candidate miRNAs that could distinguish MPO- from PR3-positive AAV-GN. The 21 out of 30 candidate miRNAs selected for validation in renal tissue samples and their specific inclusion criteria are presented in **Supplementary Table S1**.

### 3.1 Screening-based discovery of candidate renal tissue miRNAs differentially expressed in MPO- versus PR3-positive AAV-GN

The characteristics of the screening cohorts are described in detail in our previous research (9). We identified a candidate miRNA signature comprising 13 miRNAs that differentiated MPO- from PR3–positive AAV-GN. Among these, *miR-181a-5p* was already identified as capable of also distinguishing AAV-GN as a group from non-AAV GN, as well as from living donors without renal disease (9). Two other members of the *miR-181* family were identified in this screening, namely *miR-181a-2-3p* and m*iR-181d-5p*.

The results of the screening-based discovery of differentially expressed renal tissue miRNAs in MPO- compared to PR3-positive AAV-GN are illustrated in **Supplementary Figure S1**.

### 3.2 Validation of miRNA expression profiles in the renal tissue

Core clinicopathologic characteristics of the validation cohorts are presented in **Table 1**, while selected clinical and histologic variables specific to the AAV-GN group are presented in **Table 2**. See **Supplementary Results** for full clinicopathologic characteristics of the AAV-GN group.

**Table 1:** Clinicopathologic characteristics of the included cases by study groups and subgroups.

Study group	Gender ratio	Age	eGFR	DP	%IFTA	%GS	ATI
**AAV-GN**	**28:29**	**70 (18)**	**20 (24)**	**1.65 (1.89)**	**10 (28)**	**18 (31)**	**30/57**
MPO	16:17	70 (14)	19 (19)	1.6 (1.96)	10 (25)	24 (35)	16/33
PR3	12:12	66,5 (29)	26 (33)	1.65 (1.89)	5 (15)	14 (26)	14/24
**Non-AAV RD**	**13:13**	**57 (24)**	**24 (69)**	**2.5 (3.97)**	**10 (31)**	**20 (36)**	**5/26**
IRP	8:8	56 (25)	22 (57)	3.6 (3.29)	10 (25)	13 (29)	2/16
NIRP	5:5	61 (23)	23 (42)	1.57 (1.89)	10 (28)	23 (27)	3/10
**Bystander ANCA**	**5:6**	**71 (30)**	**22 (12)**	**1.83 (2)**	**30 (35)**	**24 (28)**	**4/11**
**p-value 1**	**0.968**	**0.000**	**0.012**	**0.06**	**0.127**	**0.115**	**0.017**
p-value 2	0.91	0.518	0.505	0.775	0.012	0.033	0.593
p-value 3	1.000	0.121	0.990	0.068	0.694	0.706	0.340

Gender ratio refers to male:female ratio. Numerical variables are presented as median values and IQR (in brackets). p-value 1 refers to the comparison between the three independent groups i.e., AAV-GN, non-AAV RD, and bystander ANCA (bold text), p-value 2 refers to MPO compared to PR3 subgroups, and p-value 3 refers to IRP compared to NIRP subgroups. AAV-GN, all patients with ANCA-associated glomerulonephritis; ANCA, anti-neutrophil cytoplasmic antibodies; non-AAV RD, non-AAV medical renal disease controls, either inflammatory (IRP) or non-inflammatory renal pathology (NIRP); bystander ANCA, cases with positive MPO- or PR3-ANCA without evidence of AAV; eGFR, estimated glomerular filtration rate by CKD-EPI 2021 in ml/min/1.73 m^2^; DP, measured daily proteinuria in g; %IFTA, extent of interstitial fibrosis/tubular atrophy in 5% increments; IQR, interquartile range; %GS, percentage of global glomerulosclerosis; ATI, number of cases with acute tubular injury.

**Table 2:** Clinicopathologic characteristics of the included AAV-GN cases.

Study group	BVAS^*^	AKRiS	AKRiS group	Berden	RLV	RPGN	PRS
AAV-GN	18 (10)	4 (11)	33:14:7:3	14:10:22:11	17/57	34/57	29/57
MPO	17 (9)	4 (11)	18:11:3:1	5:3:18:7	13/33	17/33	13/33
PR3	21 (9)	2 (14)	15:3:4:2	9:7:4:4	4/24	17/24	16/24
p-value	0.132	0.548	0.231	0.01	0.064	0.142	0.042

Numerical variables are presented as median values and IQR (in brackets). P-value refers to MPO compared to PR3. AAV-GN, all patients with ANCA-associated glomerulonephritis; MPO, MPO-positive AAV-GN; PR3, PR3-positive AAV-GN; BVAS, Birmingham Vasculitis Activity Score; AKRiS, ANCA Kidney Risk Score; AKRiS group, Risk groups for the AKRiS, presented as low:moderate:high: very high ratio; Berden, histologic class per ‘Histopathologic classification of ANCA-associated GN’, presented as focal:crescentic:mixed:sclerotic class ratio; IQR, interquartile range; RLV, % of cases with renal-limited vasculitis phenotype; *available for 52/57 AAV-GN cases.

#### 3.2.1 AAV-GN versus non-AAV RD group

**Figure 2:**
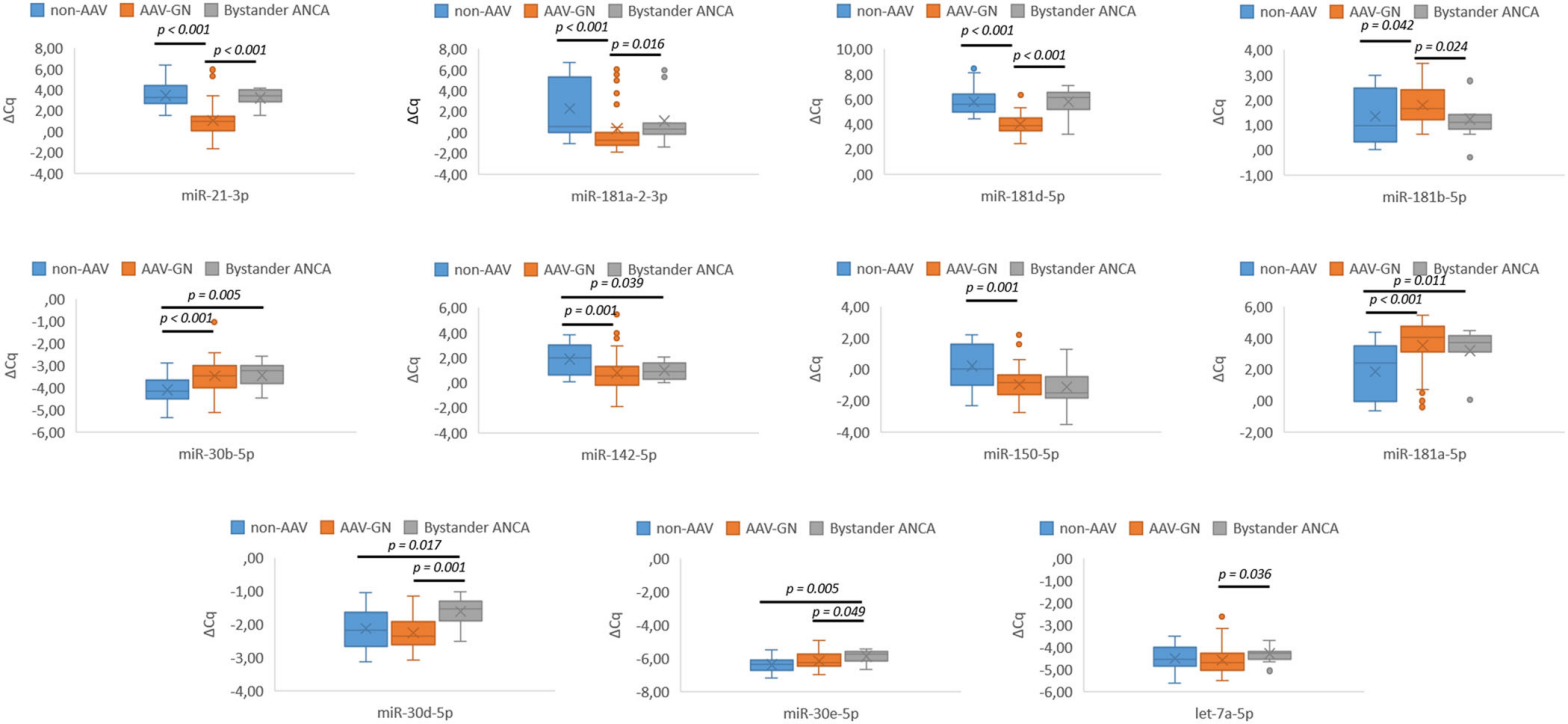
Differential expression of validated miRNAs in AAV-GN compared to non-AAV and bystander ANCA in renal tissue samples. AAV-GN, patients with ANCA-associated glomerulonephritis (n = 57); ANCA, anti-neutrophil cytoplasmic antibodies; non-AAV, non-AAV medical renal disease controls (n = 26); bystander ANCA, cases with positive MPO- or PR3-ANCA without evidence of AAV-GN (n = 11); ΔCq, delta quantitation cycle. All values were within 95% confidence interval.

Significant expression differences were established for 8 out of 21 candidate miRNAs, namely *miR-21-3p* (p < 0.001), *miR-30b-5p* (p < 0.001), *miR-142-5p* (p = 0.001), *miR-150-5p* (p = 0.001), *miR-181a-5p* (p < 0.001), *miR-181a-2-3p* (p < 0.001), *miR-181b-5p* (p = 0.042), and *miR-181d-5p* (p < 0.001) in comparisons between AAV-GN and non-AAV RD samples. **Figure 2** summarizes the results of renal tissue-based validation between AAV-GN and non-AAV RD samples.

#### 3.2.2 MPO- versus PR3-positive AAV-GN

**Figure 3:**
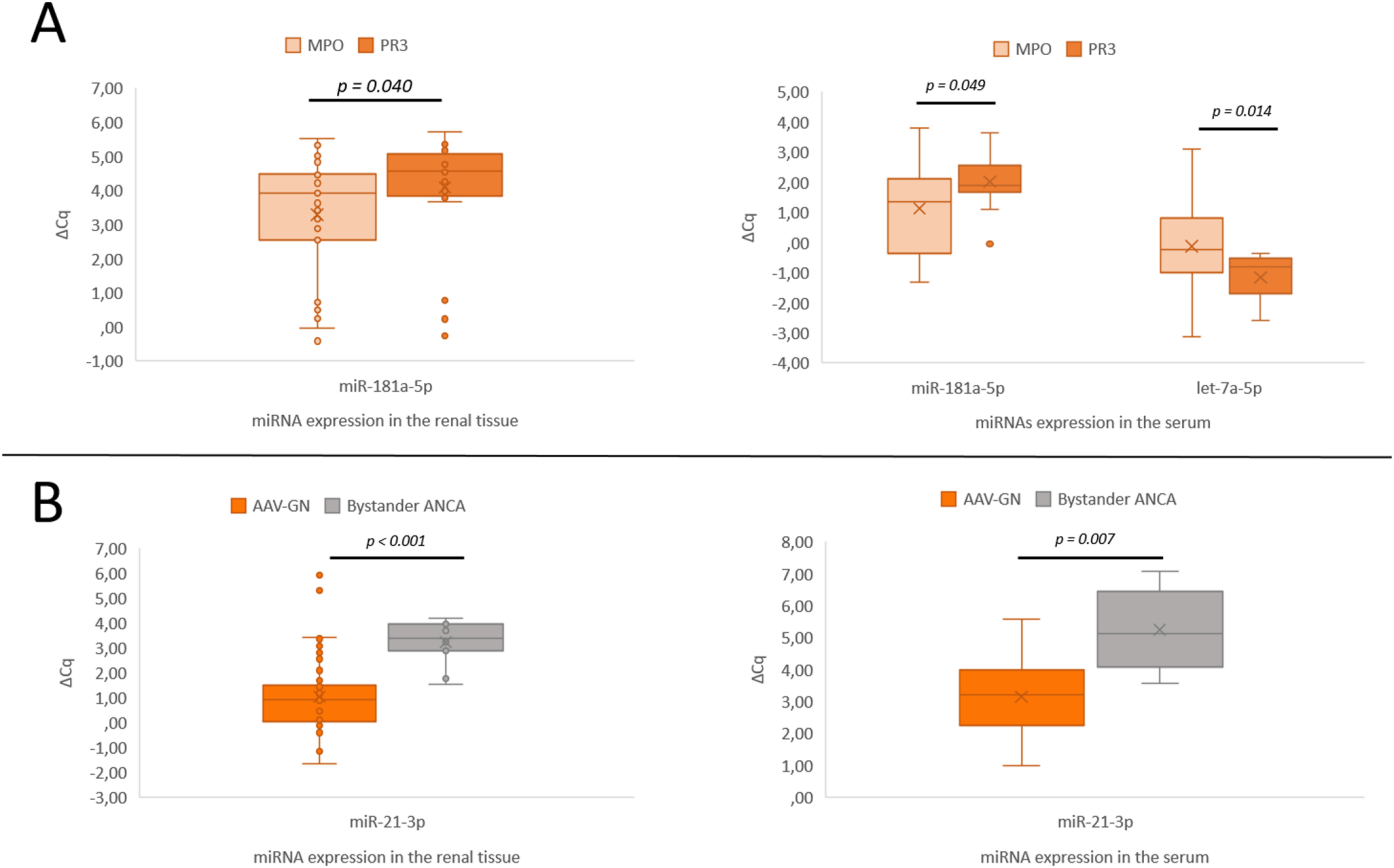
Significant expression differences of *miRNAs* in both renal tissue and serum samples. **(A)** Differential expression of *miR-181a-5p* between MPO- and PR3-positive AAV-GN. **(B)** Differential expression of *miR-21-3p* in AAV-GN compared to bystander ANCA in renal tissue and serum samples. AAV-GN, patients with ANCA-associated glomerulonephritis (n = 57 for tissue, n = 38 for serum); ANCA, anti-neutrophil cytoplasmic antibodies; MPO, MPO-positive AAV-GN (n = 33 for tissue, n = 23 for serum); PR3, PR3-positive AAV-GN (n = 24 for tissue, n = 15 for serum); bystander ANCA, cases with positive MPO- or PR3-ANCA without evidence of AAV-GN (n = 11 for tissue, n = 6 for serum); ΔCq, delta quantitation cycle. All values were within 95% confidence interval.

A significant expression difference was confirmed for *miR-181a-5p* (p = 0.04). The differential expression of any other candidate miRNA was not established. **Figure 3A** summarizes the validation results between MPO- and PR3-positive AAV-GN subgroups.

#### 3.2.3 Bystander ANCA versus AAV-GN and non-AAV RD groups

Seven candidate miRNAs, namely *miR-21-3p* (p < 0.001), *miR-30d-5p* (p = 0.001), *miR-30e-5p* (p = 0.049), *miR-181a-2-3p* (p = 0.016), *miR-181b-5p* (p = 0.024), *miR-181d-5p* (p < 0.001), and *let-7a-5p* (p = 0.036) differentiated bystander ANCA from AAV-GN and five miRNAs, namely *miR-30b-5p* (p = 0.005)*, miR-30d-5p* (p = 0.017), *miR-30e-5p* (p = 0.005), *miR-142-5p* (p = 0.039), and *miR-181a-5p* (p = 0.011) differentiated bystander ANCA from non-AAV RD samples. The expression profile of validated miRNAs in bystander ANCA samples is summarized in **Figure 2** (for miRNAs without subsequent validation in serum samples) and **Figure 3B** (for *miR-21-3p*, which translated to serum samples).

### 3.3 Expression analysis of validated miRNAs in the serum samples

Serum-based analysis included 11 candidate miRNAs with significant expression differences in the renal tissue between AAV-GN and non-AAV RD, MPO- and PR3-positive AAV-GN, and/or AAV-GN and bystander ANCA samples (validated miRNAs). The expression of validated miRNAs (*miR-21-3p, miR-30b-5p, miR-30d-5p, miR-30e-5p, miR-142-5p, miR-150-5p, miR-181a-5p, miR-181a-2-3p, miR-181b-5p, miR-181d-5p* and *let-7a-5p*) was comparatively analyzed in the serum samples of AAV, non-AAV RD, bystander ANCA and HC groups.

#### 3.3.1 AAV-GN versus non-AAV RD, and HC group

Five validated miRNAs, namely *miR-21-3p* (p < 0.001), *miR-142-5p* (p = 0.05), *miR-150-5p* (p = 0.012), *miR-181a-5p* (p = 0.002), and *miR-181d-5p* (p = 0.003) differentiated AAV-GN from non-AAV RD on serum samples. When AAV-GN was compared to HC group, the differential expressions of six validated miRNAs, namely *miR-21-3p* (p < 0.001), *miR-30d-5p* (p = 0.004), *mir-181a-5p* (p < 0.001), *mir-181a-2-3p* (p = 0.030), *miR-181d-5p* (p = 0.026), and *let-7a-5p* (p = 0.012) were significant. Conversely, when non-AAV RD group was compared to HC, the expression profile of validated miRNAs was broadly similar, except for *miR-30b-5p* (p < 0.001), *miR-142-5p* (p < 0.001), *miR-181b-5p* (p = 0.039) and *let-7a-5p* (p = 0.015).

**Figure 4:**
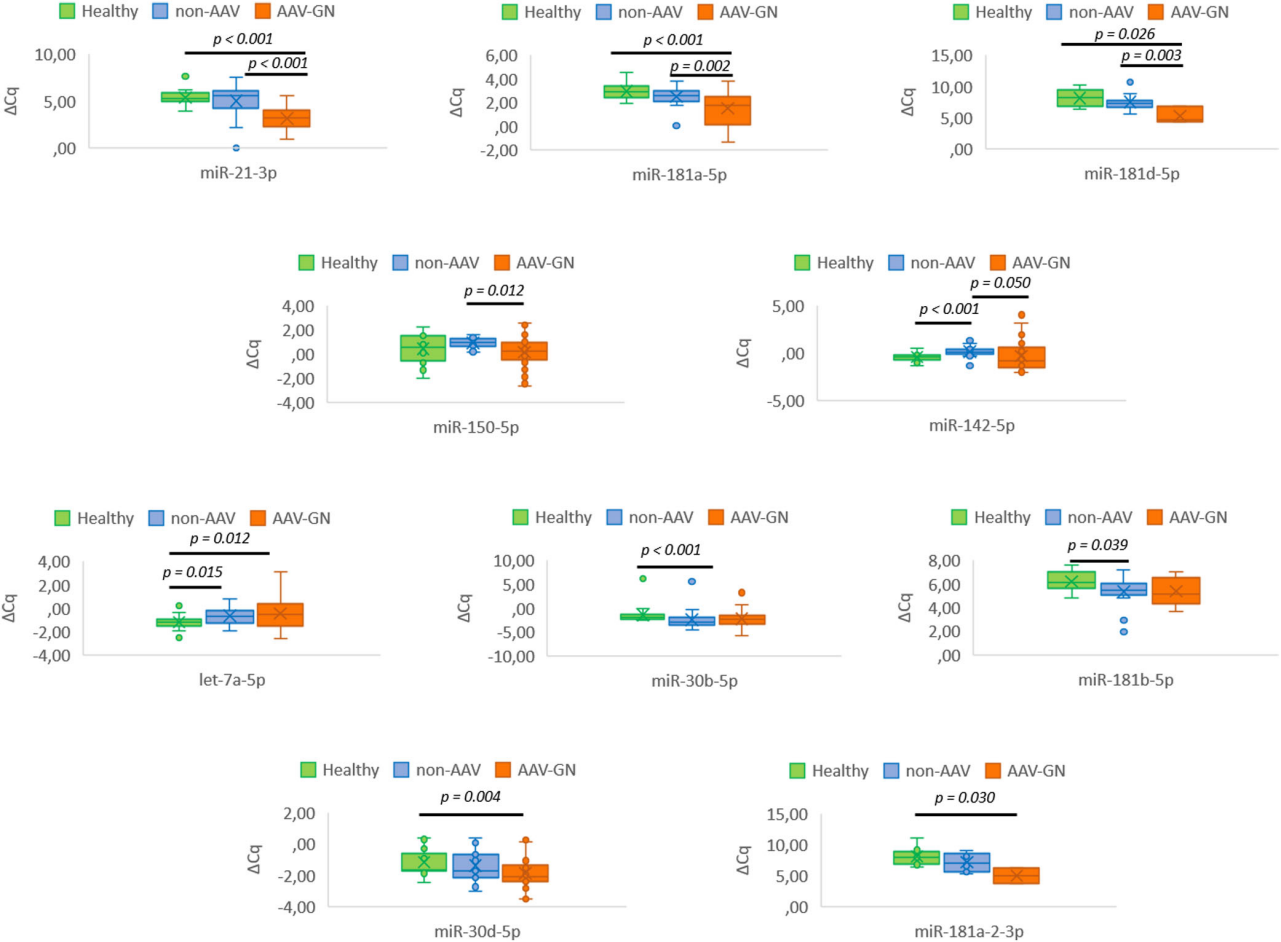
Serum-based differential expression of tissue-validated miRNAs in AAV-GN compared to non-AAV and HC groups. AAV-GN, patients with ANCA-associated glomerulonephritis (n = 38); ANCA, anti-neutrophil cytoplasmic antibodies; non-AAV, non-AAV medical renal disease controls (n = 23); Healthy, healthy serologic controls (n = 20); ΔCq, delta quantitation cycle. All values were within 95% confidence interval.

**Figure 4** summarizes the results of serum-based expression of validated miRNAs in AAV-GN compared to non-AAV RD and HC groups in box plot form.

#### 3.3.2 MPO- versus PR3-positive AAV-GN

On serum samples, significant expression difference was maintained for *miR-181a-5p* (p = 0.049), while significant difference in *let-7a-5p* (p = 0.014) expression was additionally observed in contrast to renal-tissue based analysis between MPO- and PR3-positive AAV-GN (refer to **Figure 3A** for summarized results).

#### 3.3.3 Bystander ANCA versus AAV-GN and non-AAV RD groups

Significant expression difference between bystander ANCA and AAV-GN samples was reiterated in the serum samples for *miR-21-3p* (p = 0.007, **Figure 3B**). None of the significant miRNA expression differences observed in the renal tissue translated to serum samples in comparisons between bystander ANCA and non-AAV RD samples.

### 3.4 Correlation of renal tissue and serum expression of validated miRNAs

**Figure 5:**
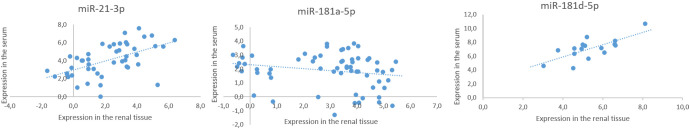
Expression correlation of validated miRNAs in matched renal tissue and serum samples. All values were within 95% confidence interval.

Significant correlations were detected in *miR-21-3p* (*ρ* = 0.491, p < 0.001), *miR-181a-5p* (*ρ* = -0.292, p = 0.020), and *miR-181d-5p* (*ρ* = 0.540, p = 0.008) expressions in corresponding renal tissue and serum samples (see **Figure 5** for summarized results). The differential expressions of some miRNAs separating AAV-GN from non-AAV RD and HC (*miR-21-3p, miR-181a-5p*, and *miR-181d-5p*) or distinguishing AAV-GN from bystander ANCA samples (*miR-21-3p*) in the serum were thus deducible from the expression differences established in the renal tissue and vice versa.

### 3.5 The ROC curve analysis

**Figure 6:**
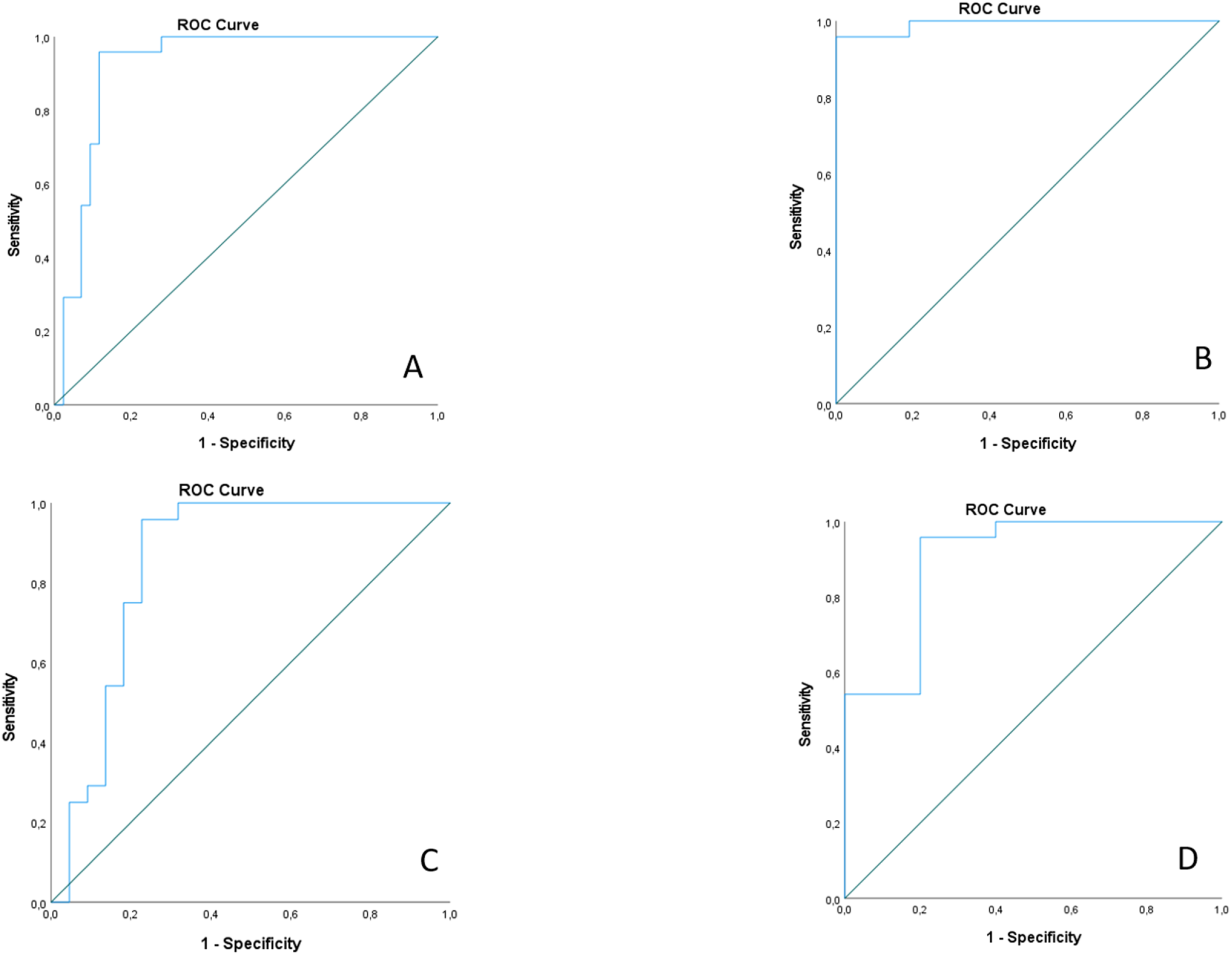
ROC curve analysis for combined *miR-21-3p* and *miR-181a-5p* serum expressions; **(A)** ROC curve for differentiation between AAV-GN and all other samples; **(B)** ROC curve for differentiation between AAV-GN and healthy controls; **(C)** ROC curve for differentiation between AAV-GN and non-AAV RD; **(D)** ROC curve for differentiation between AAV-GN and bystander ANCA. AAV-GN, patients with ANCA-associated glomerulonephritis (n = 38); ANCA, anti-neutrophil cytoplasmic antibodies; non-AAV, non-AAV medical renal disease controls (n = 23). All values were within 95% confidence interval.

We conducted ROC curve analyses using *miR-21-3p* and *miR-181a-5p* as the most promising validated serum-expressed miRNAs. Although promising, *miR-181d-5p* was excluded from the model due to reliability concerns, specifically due to an insufficient rate of detectable serum expression in the analyzed samples. The combination of *miR-21-3p* and *miR-181a-5p* still effectively distinguished AAV-GN from the other independent groups. The combined ROC model using both *miR-21-3p* and *miR-181a-5p* is illustrated in **Figure 6**. ROC models using individual miRNAs and AUC values are presented in **Supplementary Figure S2** and **Supplementary Results**, respectively.

### 3.6 Correlations and associations of validated miRNAs with established scoring systems for renal involvement in AAV-GN and clinical variables of disease activity

The increasing AKRiS correlated with the increasing renal tissue expression of *miR-142-5p, miR-150-5p* and *miR-181d-5p.* When stratified into AKRiS risk groups, similar correlations were observed with the addition of correlation with renal tissue expression of *miR-21-3p.* Comparably, increasing Berden class was associated with increasing renal tissue expressions of *miR-150-5p* and *miR-181d-5p.* The renal tissue expression of *miR-21-3p, miR-142-5p* and *miR-150-5p* correlated to %NG, and *miR-181d-5p* correlated to %C. No correlations between any validated miRNA expressions and individual histologic variables or established scoring systems were identified in the serum samples except between increasing expression of *miR-30b*-5p and decreasing %C.

BVAS correlated with the expressions of *miR-30b-5p* and *miR-181b-5p* on renal tissue and serum samples, respectively. No validated miRNA in the renal tissue or serum samples correlated to ANCA titers. Additionally, the ANCA titers did not correlate with the BVAS (p = 0.496) or AKRiS (p = 0.262) and did not associate with the Berden class (p = 0.371).

Renal tissue expression of *miR-21-3p* (p = 0.035) and *miR-142-5p* (p = 0.022) differentiated cases presenting as RPGN. No significant miRNA expression differences in the renal tissue or serum were identified comparing samples of AAV-GN with renal-limited phenotype (RLV) to those with multiorgan involvement. Conversely, increasing expression of *miR-30b-5p* in the renal tissue associated with the increasing number of affected organs and the expression of *miR-30d-5p* in the serum (p = 0.003) differentiated cases with pulmonary-renal syndrome. Pre-emptive immunosuppression did not have any effect on the characteristic miRNA signature within the AAV-GN group.

**Figure 7:**
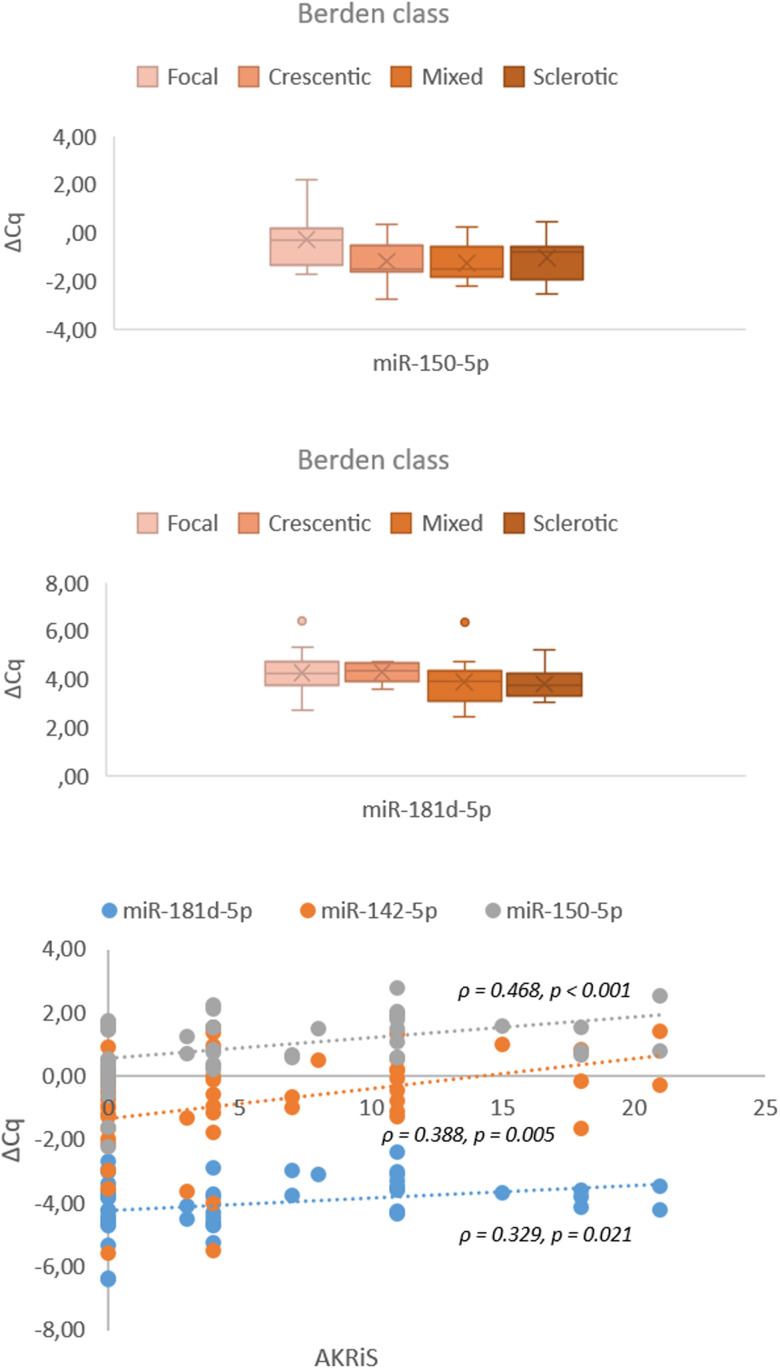
Associations and correlations of validated miRNAs in renal tissue samples to Berden class and AKRiS (n = 57). Berden class, histologic class per ‘Histopathologic classification of AAV-related GN’; AKRiS, ANCA Kidney Risk Score; ΔCq, delta quantitation cycle. All values were within 95% confidence interval.

Correlations of validated miRNAs with scoring systems and clinical variables of disease activity in renal tissue and serum samples are presented in **Table 3**. Associations and correlations of the validated miRNAs to established scoring systems for renal involvement in AAV-GN are further depicted in **Figure 7**. All correlations with biomarkers of renal function and histologic variables of chronicity are described in **Supplementary Results** and **Supplementary Table S2** including calculation of statistical power and estimation of needed sample size.

**Table 3:** Correlations and associations between the expression of validated miRNAs and scoring systems for renal involvement and clinical variables of disease activity in AAV-GN.

Variable	Renal tissue samples					Serum samples	
	*miR-30b-5p*	*miR-181d-5p*	*miR-21-3p*	*miR-142-5p*	*miR-150-5p*	*miR-30b-5p*	*miR-181b-5p*
AKRiS	/	ρ = 0.329 p = 0.021	/	ρ = 0.388 p = 0.005	ρ = 0.468 p < 0.001	/	/
AKRiS group	/	ρ = 0.411 p = 0.003	ρ = 0.327 p = 0.020	ρ = 0.405 p = 0.004	ρ = 0.384 p = 0.006	/	/
Berden class	/	ρ = 0.320 p = 0.025	/	/	ρ = 0.352 p = 0.012	/	/
%NG	/	/	ρ = -0.315 p = 0.026	ρ = -0.393 p = 0.005	ρ = -0.520 p < 0.001	/	/
%C	/	ρ = -0.287 p = 0.046	/	/	/	ρ = 0.436 p = 0.009	/
BVAS	ρ = 0.341 p = 0.019	/	/	/	/	/	ρ = -0.636 p = 0.035
No. of organs	ρ = 0.312 p = 0.027	/	/	/	/	/	ρ = -0.691 p = 0.009

AKRiS, ANCA Kidney Risk Score; AKRiS group, Risk groups for the AKRiS; Berden class, histologic class per ‘Histopathologic classification of ANCA-associated GN’; %NG, percentage of normal glomeruli; %C, percentage of cellular or fibrocellular glomerular crescents; BVAS, Birmingham Vasculitis Activity Score. Serum-based correlations are presented even though *miR-30b-5p* and *miR-181b-5p* were not statistically significant enough to distinguish between AAV-GN and non-AAV RD using serum samples.

## 4 Discussion

Our study validated significant renal tissue expression differences in 11 out of 21 candidate miRNAs selected through screening. This suggests that miRNA expression profiling in renal tissue can effectively distinguish AAV-GN from various non-AAV RD, including cases with detectable ANCA without clinical and histologic evidence of AAV-GN. Although tissue-based analysis provides valuable insights, it requires a renal biopsy to obtain sample material, which limits its applicability. Therefore, we also analyzed the expression of validated miRNAs in serum samples. Despite the inherent limitations of biofluid-based miRNA analysis (14), five validated miRNAs distinguished AAV-GN from non-AAV RD using matched serum samples. Notably, three of these five miRNAs also differentiated AAV-GN from both non-AAV RD and HC, consistent with a circulating miRNA expression profile specific to AAV. Additionally, *miR-181a-5p* also stratified AAV-GN into MPO- and PR3-positive.

Noteworthy observations apply to ‘bystander ANCA’ cases, ie. patients who underwent kidney biopsy, typically due to asymptomatic urinary abnormalities, and positive MPO- or PR3-ANCA but no histologic evidence of AAV-GN. Clinicians often struggle to distinguish between harmless (bystander) and pathogenic, disease-driving ANCAs in ANCA-positive patients. A reliable biomarker would be invaluable for guiding clinical decision-making in such cases. Comparative miRNA expression profiling in the renal tissue and serum revealed that bystander ANCA cases clustered separately from AAV-GN and non-AAV RD. While overall more aligned with non-AAV RD, bystander ANCA samples exhibited *miR-142-5p, miR-150-5p*, and *miR-181a-5p* expression patterns indistinguishable from AAV, suggesting a partial AAV-like epigenetic profile. This finding bears potential clinical implications as it raises questions about the ‘bystander’ nature of ANCAs in cases lacking clinical or histologic evidence of AAV-GN. Notably, *miR-21-3p* expression effectively differentiated AAV-GN from bystander ANCA cases in renal tissue or serum samples, highlighting its potential as a key biomarker in resolving this diagnostic uncertainty.

To refine the validated miRNA signature, the molecular data was correlated with established scoring systems for renal involvement in AAV-GN In the renal tissue, *miR-142-5p, miR-150-5p* and *miR-181d-5p* correlated with the AKRiS, while *miR-150-5p* and *miR-181d-5p* associated with the Berden classes. Accordingly, renal tissue expressions of *miR-142-5p* and *miR-150-5p* also correlated with %NG, supporting the importance of %NG in estimating risk in AAV-GN and corroborates the need for early diagnosis of kidney involvement in AAV (15–17).

Furthermore, the miRNA signature was analyzed in the context of clinical variables of disease activity. Notably, renal tissue expression of *miR-21-3p* and *miR-142-5p* differentiated AAV-GN cases presenting as RPGN, while the expression of *miR-30b-5p* in the renal tissue and the expression of *miR-181b-5p* in the serum associated with the increasing number of affected organs. Accordingly, BVAS correlated with the expressions of *miR-30b-5p* and *miR-181b-5p* on renal tissue and serum samples, respectively. Additionally, the expression of *miR-30d-5p* in the serum differentiated cases presenting with pulmonary-renal syndrome. Interestingly, no significant miRNA expression differences in the renal tissue or serum were identified comparing samples of AAV-GN labelled as RLV to those with multiorgan involvement.

Importantly, we could not directly translate any individual finding observed between variables of disease activity and validated miRNAs in the renal tissue to serum samples. This may be due to inadequate size of technically adequate serum samples, a lack of expression correlation between matched renal tissue and serum samples, or to expression levels of certain miRNAs in the serum falling below the detection threshold. Furthermore, some miRNAs may preferentially be expressed and thus detectable only in renal tissue. Conversely, aggregated miRNA expression in serum may also reflect influences from related or unrelated conditions in other organs or homeostatic mechanisms.

We would like to highlight *miR-21-3p, miR-181a-5p* and *miR-181d-5p* as the three most promising serum-expressed miRNAs. These miRNAs effectively differentiated AAV-GN from both non-AAV RD and HC cases. Moreover, the expression of *miR-21-3p* and *miR-181d-5p* differentiated the presence of RPGN and correlated with AKRiS in renal tissue samples, respectively, while *miR-181a-5p* differentiated MPO- from PR3-positive AAV-GN in renal tissue and serum samples. Furthermore, *miR-21-3p* distinguished AAV-GN from bystander ANCA cases in renal tissue and serum samples.

Importantly, *miR-21-3p, miR-181a-5p* and *miR-181d-5p* were the only validated miRNAs showing significant expression correlation between matched renal tissue and serum samples, underscoring their potential as robust noninvasive biomarkers for active AAV-GN. Additionally, ROC curve analyses demonstrated that combining serum levels of *miR-21-3p* and *miR-181a-5p* yielded a strong ability to differentiate AAV-GN from other groups. Beyond statistical analyses, functional annotations of *miR-21-3p, miR-181a-5p* and *miR-181d-5p* to biological processes implicated in AAV pathogenesis support their relevance. Namely, *miR-21-3p* and *miR-181a-5p* are implicated in altered macrophage polarization (6, 18). Indeed, alternatively activated (i.e., M2-polarized) macrophages are considered the dominant macrophage phenotype in AAV and are associated with a higher end-stage renal disease risk in AAV (4, 19).

*miR-181a-5p* also regulates the processes of B- and T-cell differentiation, namely the differentiation towards pathogenic autoantigen specific Th17 subsets in CD4+ T-cells and is involved in T-cell tolerance and selection stringency (7, 20). These processes are implicated in several autoimmune diseases, including AAV (5, 21, 22). Similarly, *miR-21-3p* overexpression promotes Th17 differentiation and activates the IL-23/IL-17 axis through MAP3K14, exacerbating inflammation in a psoriasis model (23). Elevated IL-23 and IL-17 levels were also observed in AAV (5). Interestingly, increased IL-17 levels in the context of Th17-predominant inflammation skew macrophage polarization toward the M2 phenotype (24). These insights not only align with established aspects of AAV pathogenesis but also suggest potential therapeutic implications, either by miRNA ago-/antagomirs and sponges, or by targeting implicated biologic processes with existent modalities, such as anti-IL23/IL-17 agents or abatacept (CTLA4-Ig).

It would be valuable to explore the exact function and target genes of *miR-21-3p, miR-181a-5p* and *miR-181d-5p* as the three most promising miRNAs. This can be achieved by experimentally validating the target genes of these miRNAs in appropriate cell line models. A commonly employed method involves cloning the 3’-untranslated region (3’-UTR) of the predicted miRNA target gene into a luciferase reporter construct and analyzing the change in luciferase expression as evidence of binding and regulatory activity of a particular miRNA. One such approach involves transfecting a cell line that expresses the miRNA of interest with a reporter containing either the wild-type UTR of a target gene or the UTR in which the miRNA binding site is mutated. If the construct containing the wild-type UTR shows a reduction in luciferase expression and this decrease is absent in the mutated version, this likely indicates that the endogenously expressed miRNA is capable of regulating that UTR. In this same setup, a miRNA inhibitor to block miRNA function could be used, which should lead to an increase in luciferase. Another approach is to transfect cells with both the luciferase reporter construct and increase amounts of the miRNA of interest. The advantage of introducing a miRNA is that if the miRNA really targets the binding site in the UTR, a dose-dependent effect on luciferase readout can be determined, with this response being absent with the mutated construct (25).

Several important study limitations should be acknowledged. The inherent variability of biofluid-based miRNA analysis, influenced by low isolation yields, expression variability, and the lack of standardized normalization methods, remains a challenge. This issue could be addressed by adjusting the sample volume or protocol (i.e., employing a more sensitive detection method, such as digital PCR). Additionally, the heterogeneity of renal tissue may lead to the omission or misidentification of compartment- or cell-specific miRNAs, which could be mitigated by spatial transcriptomics. Furthermore, the proposed miRNA signature relates specifically to AAV-GN and our findings may not apply to all AAV patients without renal involvement or ANCA-negative patients. Our study was also limited by small size of technically adequate samples (especially for bystander ANCA group), resulting in limited statistical power, incomplete correlation between renal tissue and serum miRNA expression for some miRNAs, and the exclusion of *miR-181d-5p* from the ROC curve analysis. Therefore, an independent validation in larger multicenter cohorts, using an alternative platform such as next-generation sequencing to reduce platform-dependent bias, is essential to confirm the proposed AAV-specific miRNA signature.

In conclusion, this study establishes *miR-21-3p, miR-181a-5p*, and *miR-181d-5p* as constituents of a circulating miRNA signature related to AAV-GN, providing a potential noninvasive biomarker for active AAV-GN in the appropriate clinical setting. By integrating renal tissue and serum miRNA expression profiles, we demonstrate that these miRNAs might effectively differentiate AAV-GN from non-AAV RD and correlate with disease activity markers. Future research, ideally as a multi-center study to increase sample sizes, should focus on refining presented findings to establish the role of identified miRNAs in the pathogenesis of AAV-GN and support the clinical application of miRNA-based biomarkers in AAV-GN diagnostics and disease monitoring.

## Data Availability

The original contributions presented in the study are included in the article/**Supplementary Material**. Further inquiries can be directed to the corresponding author.

## References

[1] Moiseev S Cohen Tervaert JW Arimura Y Bogdanos DP Csernok E Damoiseaux J. 2020 international consensus on ANCA testing beyond systemic vasculitis. Autoimmun Rev. (2020) 19:102618. doi: 10.1016/j.autrev.2020.102618, PMID: 32663621

[2] Hilhorst M van Paassen P Tervaert JWC. Limburg Renal Registry. Proteinase 3-ANCA vasculitis versus myeloperoxidase-ANCA vasculitis. J Am Soc Nephrol. (2015) 26:2314–27. doi: 10.1681/ASN.2014090903, PMID: 25956510 PMC4587702

[3] Cohen Tervaert JW. Should proteinase-3 and myeloperoxidase anti-neutrophil cytoplasmic antibody vasculitis be treated differently: part 2. Nephrol Dial Transplant. (2019) 34:384–7. doi: 10.1093/ndt/gfy406, PMID: 30668794

[4] Vegting Y Vogt L Anders H-J de Winther MPJ Bemelman FJ Hilhorst ML. Monocytes and macrophages in ANCA-associated vasculitis. Autoimmun Rev. (2021) 20:102911. doi: 10.1016/j.autrev.2021.102911, PMID: 34298153

[5] Nogueira E Hamour S Sawant D Henderson S Mansfield N Chavele K-M. Serum IL-17 and IL-23 levels and autoantigen-specific Th17 cells are elevated in patients with ANCA-associated vasculitis. Nephrol Dial Transplant. (2010) 25:2209–17. doi: 10.1093/ndt/gfp783, PMID: 20100727

[6] Huang Y Huang Y Cai Z Ferrari MW Li C Zhang T. MiR-21-3p inhibitor exerts myocardial protective effects by altering macrophage polarization state and reducing excessive mitophagy. Commun Biol. (2024) 7:1371. doi: 10.1038/s42003-024-07050-3, PMID: 39438580 PMC11496525

[7] Schaffert SA Loh C Wang S Arnold CP Axtell RC Newell EW. Mir-181a-1/b-1 modulates tolerance through opposing activities in selection and peripheral T cell function. J Immunol. (2015) 195:1470–9. doi: 10.4049/jimmunol.1401587, PMID: 26163591 PMC4763610

[8] Pauley KM Cha S Chan EKL. MicroRNA in autoimmunity and autoimmune diseases. J Autoimmun. (2009) 32:189–94. doi: 10.1016/j.jaut.2009.02.012, PMID: 19303254 PMC2717629

[9] Bošnjak M Večerić-Haler Ž Boštjančič E Kojc N. Renal tissue miRNA expression profiles in ANCA-associated vasculitis-A comparative analysis. Int J Mol Sci. (2021) 23:105. doi: 10.3390/ijms23010105, PMID: 35008531 PMC8745125

[10] Mukhtyar C Lee R Brown D Carruthers D Dasgupta B Dubey S. Modification and validation of the birmingham vasculitis activity score (version 3). Ann Rheum Dis. (2009) 68:1827–32. doi: 10.1136/ard.2008.101279, PMID: 19054820

[11] Berden AE Ferrario F Hagen EC Jayne DR Jennette JC Joh K. Histopathologic classification of ANCA-associated glomerulonephritis. J Am Soc Nephrol. (2010) 21:1628–36. doi: 10.1681/ASN.2010050477, PMID: 20616173

[12] Bate S McGovern D Costigliolo F Tan PG Kratky V Scott J. The improved kidney risk score in ANCA-associated vasculitis for clinical practice and trials. J Am Soc Nephrol. (2024) 35:335–46. doi: 10.1681/ASN.0000000000000274, PMID: 38082490 PMC10914211

[13] Latham GJ. Normalization of microRNA quantitative RT-PCR data in reduced scale experimental designs. Methods Mol Biol. (2010) 667:19–31. doi: 10.1007/978-1-60761-811-9_2, PMID: 20827524

[14] Connor KL Denby L. MicroRNAs as non-invasive biomarkers of renal disease. Nephrol Dial Transplant. (2021) 36:428–9. doi: 10.1093/ndt/gfz183, PMID: 31539062 PMC7898020

[15] Brix SR Noriega M Tennstedt P Vettorazzi E Busch M Nitschke M. Development and validation of a renal risk score in ANCA-associated glomerulonephritis. Kidney Int. (2018) 94:1177–88. doi: 10.1016/j.kint.2018.07.020, PMID: 30385041

[16] Sachez-Alamo B Moi L Bajema I Berden A Flossmann O Hruskova Z. Long-term outcome of kidney function in patients with ANCA-associated vasculitis. Nephrol Dial Transplant. (2024) 39:1483–93. doi: 10.1093/ndt/gfae018, PMID: 38268409 PMC11361807

[17] Villacorta J Diaz-Crespo F Guerrero C Acevedo M Cavero T Fernandez-Juarez G. Long-term validation of the renal risk score for vasculitis in a Southern European population. Clin Kidney J. (2021) 14:220–5. doi: 10.1093/ckj/sfaa073, PMID: 33564422 PMC7857782

[18] Jiang M Dai J Yin M Jiang C Ren M Tian L. LncRNA MEG8 sponging miR-181a-5p contributes to M1 macrophage polarization by regulating SHP2 expression in Henoch-Schonlein purpura rats. Ann Med. (2021) 53:1576–88. doi: 10.1080/07853890.2021.1969033, PMID: 34477472 PMC8425717

[19] Bitton L Vandenbussche C Wayolle N Gibier J-B Cordonnier C Verine J. Tubulointerstitial damage and interstitial immune cell phenotypes are useful predictors for renal survival and relapse in antineutrophil cytoplasmic antibody-associated vasculitis. J Nephrol. (2020) 33:771–81. doi: 10.1007/s40620-019-00695-y, PMID: 31916228

[20] Sun X Sit A Feinberg MW. Role of miR-181 family in regulating vascular inflammation and immunity. Trends Cardiovasc Med. (2014) 24:105–12. doi: 10.1016/j.tcm.2013.09.002, PMID: 24183793 PMC3943593

[21] Pfeifle R Rothe T Ipseiz N Scherer HU Culemann S Harre U. Regulation of autoantibody activity by the IL-23-TH17 axis determines the onset of autoimmune disease. Nat Immunol. (2017) 18:104–13. doi: 10.1038/ni.3579, PMID: 27820809 PMC5164937

[22] Krebs CF Panzer U. Plasticity and heterogeneity of Th17 in immune-mediated kidney diseases. J Autoimmun. (2018) 87:61–8. doi: 10.1016/j.jaut.2017.12.005, PMID: 29275837

[23] Abdallah F Henriet E Suet A Arar A Clemençon R Malinge J-M. MiR-21-3p/IL-22 axes are major drivers of psoriasis pathogenesis by modulating keratinocytes proliferation-survival balance and inflammatory response. Cells. (2021) 10:2547. doi: 10.3390/cells10102547, PMID: 34685526 PMC8534095

[24] Zizzo G Cohen PL. IL-17 stimulates differentiation of human anti-inflammatory macrophages and phagocytosis of apoptotic neutrophils in response to IL-10 and glucocorticoids. J Immunol. (2013) 190:5237–46. doi: 10.4049/jimmunol.1203017, PMID: 23596310 PMC3677729

[25] van Rooij E. The art of microRNA research. Circ Res. (2011) 108:219–34. doi: 10.1161/CIRCRESAHA.110.227496, PMID: 21252150

