# Supplementary figures and images for: Circulating miRNAs as potential non-invasive biomarkers for ANCA-associated glomerulonephritis
Source: Front Immunol. 2025 Jul 17;16:1599043. doi: 10.3389/fimmu.2025.1599043 (PMC12310630; doi:10.3389/fimmu.2025.1599043)

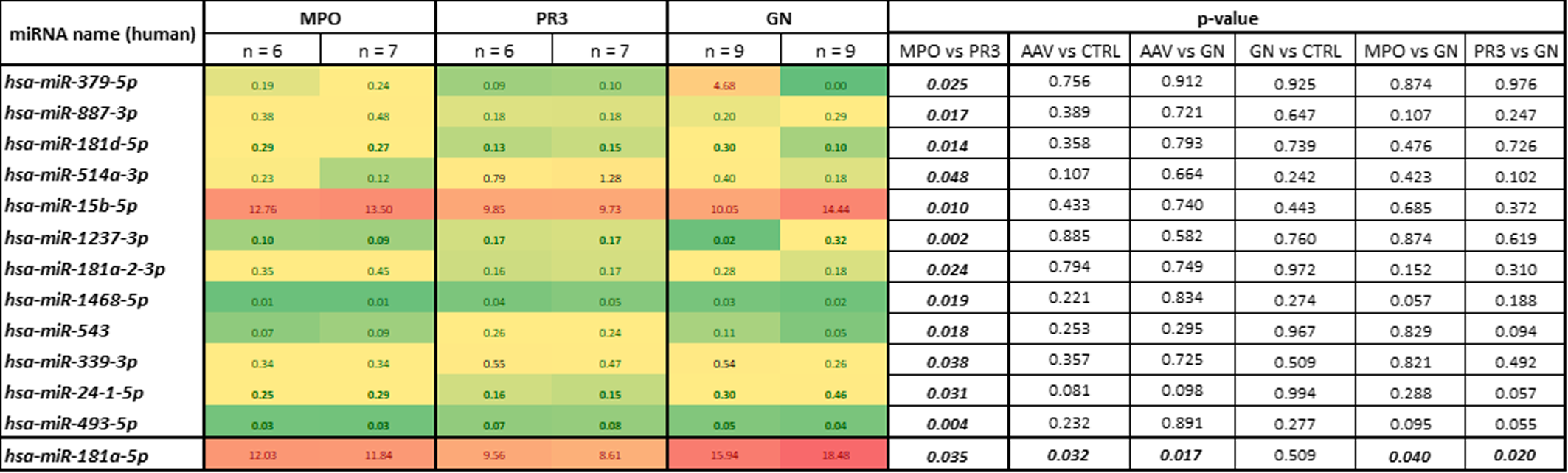

Supplement: Supplementary file 1 [file Image1.tif]

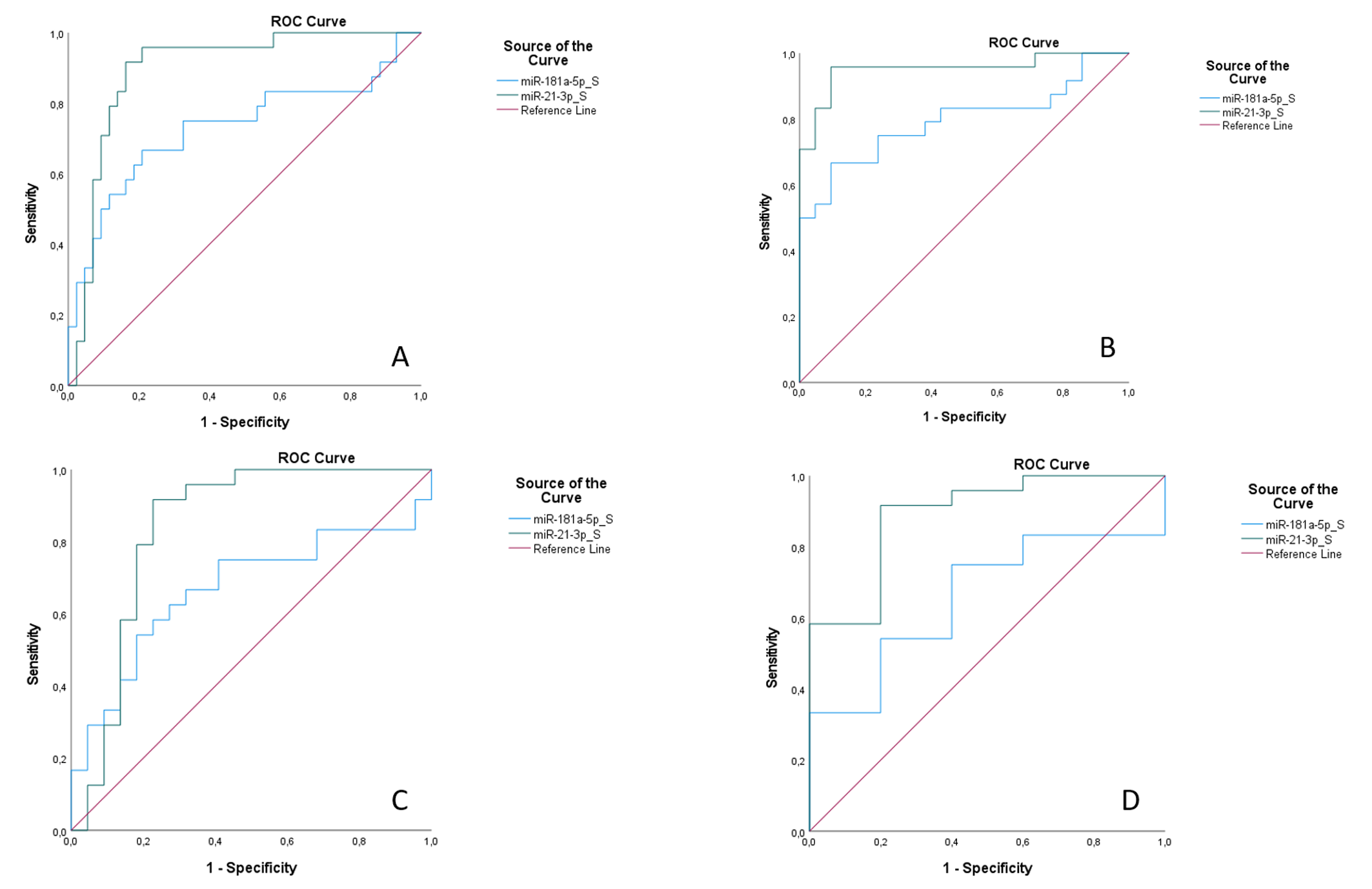

Supplement: Supplementary file 2 [file Image2.tif]
