# Supplementary material for: Circulating miRNAs as potential non-invasive biomarkers for ANCA-associated glomerulonephritis
Source: Front Immunol. 2025 Jul 17;16:1599043. doi: 10.3389/fimmu.2025.1599043 (PMC12310630; doi:10.3389/fimmu.2025.1599043)
Supplement: Supplementary file 3 [file SupplementaryFile1.docx]

Supplementary Material

# Supplementary Materials & Methods

## Detailed clinicopathologic characteristics of the validation cohorts

The AAV-GN group included 33 MPO- and 24 PR3- positive AAV-GN cases. In the AAV-GN group, 3/57 cases had concurrent IgA nephropathy, while the remaining 54 cases were ‘pure’ AAV-GN. The non-AAV RD group included 16 ‘inflammatory renal pathology’ (IRP) and 10 ‘non-inflammatory renal pathology’ (NIRP) cases. Specifically, the IRP subgroup included 6 IgA nephropathy (IgAN), 8 membranous nephropathy and 2 anti-GBM glomerulonephritis cases. The NIRP subgroup included 3 thin glomerular basement membrane nephropathy, 1 focal segmental glomerulosclerosis (FSGS), 2 focal non-specific chronic change, 1 diabetic nephropathy, 2 thrombotic microangiopathy and 1 minimal change disease case. The bystander ANCA group included 5 MPO-ANCA positive cases (1 IgAN, 1 infection-related glomerulonephritis, 1 FSGS, 1 focal non-specific chronic change and 1 diabetic nephropathy) and 6 PR3-ANCA positive cases (4 IgAN, 1 FSGS and 1 C3 glomerulonephritis).

## ANCA testing

IgG ANCA were tested by indirect immunofluorescence on ethanol-fixed normal human leukocytes and scored as cytoplasmic ANCA (c-ANCA) or perinuclear ANCA (p-ANCA) pattern. ANCA antigen specificities were determined using a commercial ELISA kit (Wieslab AB, Malmö, Sweden). With this kit, the positive cut-off levels were set at 8 IU/ml for MPO-ANCA and 6 IU/ml for PR3-ANCA.

## Total RNA isolation from renal tissue samples

Specifically, tissue was de-waxed using 300 µL of mineral oil followed by protein digestion step overnight, in accordance with the Maxwell® RSC RNA FFPE kit instructions. Following this, lysis buffer, lysis enhancer and proteinase K were added to the aqueous solution of digested samples. The entire volume was then transferred into the Maxwell® RSC miRNA tissue kit cartridge for total RNA isolation, which included a DNase digestion step. Elution was performed in 60 µL of nuclease-free water (Promega, Madison, WI 53711-5399, USA) and the eluate was stored at – 80°C. Prior to further analysis, RNA purity was assessed by measuring A260/280 and A260/230 using a NanoDrop-One (Applied Biosystems; ThermoFisher Scientific, Foster City, CA, USA) and RNA yield was measured by high-sensitivity RNA assay (Qubit 3.0 Fluorometer, Applied Biosystems; ThermoFisher Scientific, Foster City, CA, USA).

## Total RNA isolation from serum samples

Elution was performed in 20 µL of nuclease-free water and the eluate was stored at – 80°C. Spike-in RNAs, namely *UniSp2, UniSp4 and UniSp5* (Qiagen, Hilden, Germany), were used to verify the technical success of the isolation procedure.

## Reverse transcription into cDNA

Using the miRCURY LNA RT Kit (Qiagen, Hilden, Germany), reverse transcription (RT) was subsequently conducted according to the manufacturer’s instructions, including the addition of the spike-in *UniSp6* (Qiagen, Hilden, Germany) to verify RT technical success.
For tissue samples, 10 μl reaction master mix contained 10 ng total RNA. For serum samples, 10 μl reaction master mix contained 2 μl of isolated RNA.

## Selection of candidate miRNAs for validation in renal tissue and serum samples

Candidate miRNAs from the two screening phases with Cq values ≤ 30 were selected for validation. Additionally, candidate miRNAs belonging to the same miRNA family were included regardless of their Cq values, if there were at least 2 candidate miRNAs within that family. Based on these criteria, we included 10 out of 17 ‘AAV-GN-specific’ candidate miRNAs and 4 out of 13 ‘MPO/PR3 classifying’ candidate miRNAs for validation. Additionally, seven other miRNAs from the three most represented miRNA families that had not been statistically selected as ‘AAV-GN specific’ or ‘MPO/PR3 classifying’ through screening were also included for validation. The 21 miRNAs selected for validation in renal tissue samples and their specific inclusion criteria are presented in Supplementary Table S1.

## Selection of reference genes

The set of reference genes (RGs) for normalizing miRNA expression in renal tissue samples was selected from the RG pool used in the preceding screening phase. Briefly, we analyzed the expression of RGs based on two criteria: standard deviations between all sample pools < 0.5 and no statistically significant differences in expression between the independent groups. Four RG miRNAs with the smallest standard deviation between pools, namely *miR-23a-3p, miR-25-3p, miR-28-5p* and *miR-185-5p*, were selected.

For the serum samples, *miR-103a-3p, miR-191-5p*, and *miR-423* were used as RGs according to the manufacturer’s instruction. The presence of sample hemolysis was excluded by quantifying the expression of *miR-23a* and *miR-451a* (control miRNAs). The technical success of the miRNA isolation procedure from serum was confirmed by quantifying the expression of *UniSp2, UniSp4* and *UniSp5* spike-ins.

## Quantification of the selected miRNAs, RGs, and control miRNAs

Prior to qPCR, the amplification efficiency relative to RGs for each analyzed miRNA was determined in pooled RNA isolates followed by RT and using its 4-fold dilutions. Each such reaction was performed in a triplicate.
The RT yield from tissue samples was diluted 60-fold, while RT from serum samples was diluted 30-fold. In both cases, 3 μl was used in 10 μl qPCR reaction master mix, in accordance with the manufacturer’s instructions. All qPCR reactions were performed in duplicate on the QuantStudio 7 Pro platform (Thermo Fisher Scientific, Foster City, CA, USA) according to the manufacturer’s instructions. The signal was collected at the endpoint of every cycle. To ascertain the specificity of the qPCR products, melting curve analysis was conducted using a ramping rate of 0.075°C/1s in the 60–95°C range.

## Statistical analysis of correlations and ROC curve analysis

For all the correlations between expression of miRNA in matched tissue and serum samples, as well as correlations and associations between expressions of miRNAs and clinical and histologic variables, we calculated the Spearman’s rank correlation coefficient (Spearman’s rho). The receiver operating characteristic (ROC) curve analysis included the most promising validated serum-expressed miRNAs, i.e. miRNAs capable of differentiating AAV-GN from non-AAV RD, HC and/or bystander ANCA serum samples that demonstrated both expression correlation in paired renal tissue and serum samples and representable serum expression, i.e. detectable serum expression of miRNA across most serum samples.

# Supplementary Results

## Full characteristics of the AAV-GN cohort

The percentage of normal or crescentic glomeruli (cellular or fibrocellular crescents) did not vary significantly between the MPO- and PR3-positive AAV-GN (p = 0.716 and p = 0.132, respectively). Extraglomerular small vessel vasculitis was found in 11/57 (as arteritis in 5 and as necrotizing peritubular capillaritis in 6). Pre-emptive immunosuppression was administered to 18 AAV-GN cases prior to renal biopsy, and 13 cases were treated with plasmaphereses.
The quantitative analysis of ANCA titers was restricted to 52/57 AAV-GN cases. For MPO-positive AAV-GN, titers ranged from 7 to 200 IU/ml, with a median of 130 IU/ml, while for PR3-positive AAV-GN, titers ranged from 10 to 200 IU/ml, with a median of 44 IU/ml.

## Correlations of validated miRNAs with biomarkers of renal function and histologic variables of chronicity in AAV-GN, non-AAV RD and bystander ANCA

Correlations were observed between eGFR and the expression of *miR-21-3p* (ρ = 0.389, p < 0.001; ρ = 0.490, p < 0.001) and *miR-181a-5p* (ρ = -0.215, p < 0.041; ρ = 0.323, p = 0.004) in both renal tissue and serum samples, respectively. Several other validated miRNAs correlated to eGFR in either renal tissue samples (*miR-30b-5p, miR-30d-5p, miR-30e-5p, miR-181d-5p, miR-142-5p* and *miR-150-5p*) or serum samples (*miR-181a-2-3p, miR-181b-5p* and *let-7a-5p)* only. Similarly, ATI was associated with the expressions of *miR-21-3p,  miR-142-5p, miR-150-5p* and *miR-181a-5p* in both renal tissue and serum samples, whereas other miRNAs correlated to ATI in either renal tissue (*miR-30b-5p* and *miR-30e-5p*) or serum samples (*miR-30d-5p,* *miR-181a-2-3p, miR-181d-5p)*. DP correlated with the renal tissue expression of *let-7a-5p* and serum expression of *miR-181d-5p.* 
In the renal tissue, the expressions of *miR-30e-5p, miR-181a-2-3p, miR-181d-5p, miR-142-5p* and *miR-150-5p* correlated to %GS, and *miR-30d-5p* and *miR-150-5p* correlated to %IFTA. No such correlations were identified in the serum samples with any serum-expressed validated miRNA. 
Correlations of validated miRNAs with nonspecific variables (biomarkers of renal function and histologic variables of chronicity) in renal tissue and serum samples are summarized in Supplementary Table S1.  Of note, the ΔCq value is calculated as the difference between the quantification cycle (Cq) of the gene of interest (GOI) and the geometric mean of the reference genes (RGs), expressed as ΔCq = Cq__GOI_ – Cq__GEOMEAN_RG_. A negative ΔCq value indicates higher expression of the miRNA, corresponding to a lower Cq value for the GOI relative to the reference genes. Conversely, a positive ΔCq value reflects lower miRNA expression, as evidenced by a higher Cq value for the GOI compared to the geometric mean of the reference genes. It is important to note that, due to this inverse relationship, negative correlations in ΔCq values correspond to positive correlations in actual expression levels, and vice versa.

## AUC values for individual comparisons through ROC curve analysis

AUC values for the comparisons using a combination of *miR-21-3p* and *miR-181a-5p* were as follows: AUC of 0.920 (p < 0.001) for differentiation between AAV-GN and other groups combined; AUC of 0.852 (p < 0.001) for AAV-GN versus non-AAV RD; AUC of 0.900 (p = 0.006) for AAV-GN versus bystander ANCA; and AUC of 0.992 (p < 0.001) for AAV-GN versus HC.

# Supplementary Figure legends

**Supplementary Fig. S1.** Heat map of differentially expressed renal tissue miRNAs in MPO-positive compared to PR3-positive AAV-GN discovered through screening. Legend: n = number of samples per pool; AAV-GN, patients with ANCA-associated glomerulonephritis; GN, control subjects with non-AAV glomerulonephritis; CTRL, control subjects without evidence of medical renal disease or with isolated microhematuria; MPO, MPO-positive AAV-GN; PR3, PR3-positive AAV-GN.

**Supplementary Fig. S2.** ROC curve analysis for individual serum-expressed miRNAs. A. AAV-GN versus all other samples. B. AAV-GN versus healthy controls. C. AAV-GN versus non-AAV RD. D. AAV-GN versus bystander ANCA. Legend: AAV-GN, patients with ANCA-associated glomerulonephritis; non-AAV, non-AAV medical renal disease controls.

# Supplementary Table legends

**Supplementary Table S2. Full correlations between analysed miRNA expression and histopathological analysis.** Note that due to the nature of ΔCq calculation (see explanation in section Supplementary results 2.2), negative correlations in ΔCq values correspond to positive correlations in actual expression levels, and vice versa. In the ‘AKRiS, PRS, RPGN’ sheet the ‘miR-NAME_S’ signifies serum expression of that miRNA and ‘miR-NAME’ corresponds to renal expression of that miRNA.
