# Supplementary material for: Circulating miRNAs as potential non-invasive biomarkers for ANCA-associated glomerulonephritis
Source: Front Immunol. 2025 Jul 17;16:1599043. doi: 10.3389/fimmu.2025.1599043 (PMC12310630; doi:10.3389/fimmu.2025.1599043)
Supplement: Supplementary file 4 [file Table1.docx]

**Supplementary Table S1.** Candidate screening-based miRNAs for validation in renal tissue samples and their inclusion criteria. Legend: AAV-GN, patients with ANCA-associated glomerulonephritis; MPO, MPO-positive AAV-GN; PR3, PR3-positive AAV-GN; GN, control subjects with non-AAV-GN glomerulonephritis; CTRL, control subjects without evidence of medical renal disease or with isolated microhematuria; AAV-GN specific, miRNAs that potentially distinguish AAV-GN as a group from GN and CTRL; AAV-GN classifying, miRNAs that potentially classify AAV-GN into MPO- and PR3-positive; RGs, reference gene miRNAs; Cq, threshold PCR quantitation cycle; Family, candidate miRNAs that were included from the most represented miRNA families regardless of Cq; StDev, standard deviation.

|  | miRNA (human) | CTRL pool 1 | CTRL pool 2 | MPO pool 1 | MPO pool 2 | PR3 pool 1 | PR3 pool 2 | GN pool 1 | GN pool 2 | Criteria | Catalogue number |
| --- | --- | --- | --- | --- | --- | --- | --- | --- | --- | --- | --- |
| *AAV-GN specific* | *hsa-miR-30c-5p* | 21.96 | 22.41 | 23.31 | 23.40 | 23.76 | 23.58 | 22.98 | 23.41 | Cq < 30 | YP00204783 |
|  | *hsa-miR-204-5p* | 22.86 | 23.31 | 24.44 | 24.56 | 25.00 | 24.52 | 24.15 | 24.30 | Cq < 30 | YP00206072 |
|  | *hsa-miR-30b-5p* | 22.63 | 23.23 | 23.62 | 24.38 | 24.47 | 24.27 | 23.74 | 23.91 | Cq < 30 | YP00204765 |
|  | *hsa-miR-150-5p* | 27.99 | 26.37 | 25.28 | 25.08 | 26.00 | 25.79 | 26.19 | 26.60 | Cq < 30 | YP00204660 |
|  | *hsa-let-7a-5p* | 22.79 | 23.19 | 23.33 | 24.33 | 24.31 | 24.15 | 23.69 | 23.67 | Cq < 30 | YP00205727 |
|  | *hsa-miR-142-5p* | 30.38 | 30.26 | 28.51 | 30.09 | 29.62 | 29.41 | 30.07 | 30.27 | Cq < 30 | YP00204722 |
|  | *hsa-miR-21-3p* | 29.48 | 30.24 | 28.39 | 28.93 | 28.67 | 28.92 | 29.58 | 30.28 | Cq < 30 | YP00204302 |
|  | *hsa-miR-181a-5p* | 26.25 | 26.54 | 26.72 | 27.40 | 27.69 | 27.83 | 26.82 | 26.88 | Cq < 30 | YP00206081 |
|  | *hsa-miR-24-2-5p* | 32.32 | 32.71 | 31.63 | 31.49 | 32.07 | 33.01 | 34.79 | 33.70 | Family | YP00204457 |
|  | *hsa-miR-30a-3p* | 26.17 | 26.13 | 27.35 | 27.61 | 27.66 | 27.22 | 26.85 | 27.28 | Cq < 30 | YP00204187 |
| *AAV-GN classifying* | *hsa-miR-181d-5p* | 33.26 | 32.90 | 32.08 | 32.85 | 33.88 | 33.66 | 32.54 | 34.38 | Family | YP00204789 |
|  | *hsa-miR-15b-5p* | 26.72 | 27.07 | 26.63 | 27.21 | 27.65 | 27.66 | 27.48 | 27.24 | Cq < 30 | YP00204243 |
|  | *hsa-miR-181a-2-3p* | 32.70 | 32.07 | 31.84 | 32.10 | 33.60 | 33.50 | 32.67 | 33.58 | Family | YP00204142 |
|  | *hsa-miR-24-1-5p* | 31.63 | 31.68 | 32.31 | 32.73 | 33.55 | 33.72 | 32.53 | 32.20 | Family | YP00204357 |
| *RGs* | *hsa-miR-185-5p* | 28.39 | 28.51 | 27.88 | 28.39 | 28.53 | 28.73 | 28.62 | 28.72 | StDev = 0.027 | YP0020**6037** |
|  | *hsa-miR-25-3p* | 26.89 | 27.25 | 27.09 | 26.69 | 27.54 | 27.19 | 26.93 | 27.36 | StDev = 0.027 | YP00204361 |
|  | *hsa-miR-23a-3p* | 23.86 | 24.31 | 23.87 | 23.78 | 23.89 | 24.13 | 24.17 | 24.57 | StDev = 0.027 | YP00204772 |
|  | *hsa-miR-28-5p* | 28.61 | 28.96 | 29.01 | 28.57 | 28.74 | 28.88 | 28.73 | 29.00 | StDev = 0.017 | YP00204322 |
| *Lead miRNA strand of represented miRNA families* | *hsa-miR-30a-5p* | 21.73 | 22.23 | 22.87 | 23.41 | 23.42 | 23.16 | 23.12 | 23.37 | Family | YP00205695 |
|  | *hsa-miR-30d-5p* | 22.96 | 23.55 | 24.09 | 24.52 | 24.32 | 24.55 | 24.34 | 24.58 | Family | YP00206047 |
|  | *hsa-miR-30e-3p* | 25.97 | 26.24 | 27.27 | 27.42 | 28.22 | 27.19 | 26.83 | 27.25 | Family | YP00204714 |
|  | *hsa-miR-30e-5p* | 25.18 | 25.57 | 26.49 | 26.82 | 26.89 | 26.54 | 26.37 | 26.60 | Family | YP00204410 |
|  | *hsa-miR-181b-5p* | 28.25 | 28.66 | 28.62 | 28.49 | 29.47 | 29.48 | 28.95 | 29.33 | Family | YP00204530 |
|  | *hsa-miR-181c-5p* | 33.29 | 33.70 | 33.06 | 34.62 | 34.65 | 34.84 | 34.70 | 34.74 | Family | YP00204683 |
|  | *hsa-miR-24-3p* | 23.65 | 24.06 | 23.86 | 24.07 | 24.78 | 24.44 | 23.92 | 24.24 | Family | YP00204260 |
